# Influence of Angiotensin-converting Enzyme Insertion/Deletion Gene Polymorphism in Progression of Chagas Heart Disease

**DOI:** 10.1590/0037-8682-0488-2019

**Published:** 2020-07-03

**Authors:** Silvia Marinho Martins Alves, Lúcia Elena Alvarado-Arnês, Maria da Glória Aureliano de Melo Cavalcanti, Cristina de Fátima Velloso Carrazzone, Antônio Guilherme Fonseca Pacheco, Camila Sarteschi, Milton Ozorio Moraes, Wilson Alves de Oliveira, Carolina de Araújo Medeiros, Fernanda Gallinaro Pessoa, Charles Mady, Joseli Lannes-Vieira, Felix José Alvarez Ramires

**Affiliations:** 1 Instituto do Coração (InCor), Hospital das Clínicas HCFMUSP, Faculdade de Medicina, Universidade de São Paulo, São Paulo, SP, Brasil.; 2 Fundação Oswaldo Cruz, Instituto Oswaldo Cruz, Laboratório de Biologia das Interações, Rio de Janeiro, RJ, Brasil.; 3 Ambulatório de Doença de Chagas e Insuficiência Cardíaca, Pronto Socorro Cardiológico de Pernambuco (PROCAPE)/UPE, Recife, PE, Brasil.; 4 Fundação Oswaldo Cruz, Instituto Oswaldo Cruz, Laboratório de Hanseníase, Rio de Janeiro, RJ, Brasil.; 5 Fundação Oswaldo Cruz, Programa de Computação Científica, Rio de Janeiro, RJ, Brasil.; 6 Realcor - Real Hospital Português de Beneficência, Recife, PE, Brasil.

**Keywords:** Chagas disease, *ACE* I/D polymorphism, Cardiomyopathy, Heart failure

## Abstract

**INTRODUCTION:**

Chagas disease (CD) is a neglected disease caused by the parasite *Trypanosoma cruzi*. One-third of infected patients will develop the cardiac form, which may progress to heart failure (HF). However, the factors that determine disease progression remain unclear. Increased angiotensin II activity is a key player in the pathophysiology of HF. A functional polymorphism of the angiotensin-converting enzyme (ACE) gene is associated with plasma enzyme activity. In CD, ACE inhibitors have beneficial effects supporting the use of this treatment in chagasic cardiomyopathy.

**METHODS:**

We evaluated the association of ACE I/D polymorphism with HF, performing a case-control study encompassing 343 patients with positive serology for CD staged as non-cardiomyopathy (stage A; 100), mild (stage B1; 144), and severe (stage C; 99) forms of Chagas heart disease. For *ACE* I/D genotyping by PCR, groups were compared using unconditional logistic regression analysis and adjusted for nongenetic covariates: age, sex, and trypanocidal treatment.

**RESULTS:**

A marginal, but not significant (p=0.06) higher prevalence of *ACE* I/D polymorphism was observed in patients in stage C compared with patients in stage A. Patients in stage C (CD with HF), were compared with patients in stages A and B1 combined into one group (CD without HF); DD genotype/D carriers were prevalent in the HF patients (OR = 2; CI = 1.013.96; p = 0.04).

**CONCLUSIONS:**

Our results of this cohort study, comprising a population from the Northeast region of Brazil, suggest that *ACE* I/D polymorphism is more prevalent in the cardiac form of Chagas disease with HF**.**

## INTRODUCTION

Chagas disease (CD) is a neglected tropical disease caused by the intracellular protozoan parasite *Trypanosoma cruzi*. The World Health Organization estimates that 8 to 9 million people are infected in Latin America, where CD is endemic^1^. In recent decades, migration has changed the epidemiological profile of CD, and infected people are also found in North America, Europe, and Asia^1^ ^-^ ^4^. Many countries in Latin America received international certification for the elimination of Chagas disease transmission by the main vector *Triatoma infestans*, which reflected a significant reduction in the incidence of acute cases. However, there is persistent transmission in some of these countries, such as the North and Northeast regions of Brazil, possibly linked to transmission by autochthonous species of the vector, such as *Triatoma brasiliensis,* and food-born oral transmission^5^. The oral transmission is responsible for higher acute mortality and a worse prognosis. Importantly, approximately 10 million chronically infected patients worldwide remain untreated and require medical follow-up^1^ ^,^ ^6^. 

The acute phase of CD is predominantly asymptomatic. However, in the chronic phase, the disease evolves to a broad spectrum of presentations, ranging from the indeterminate form, with a favorable evolution, to the most severe cardiac form in 20 % to 30 % of the infected individuals^6^ ^,^ ^7^. Further, the cardiac form of CD may be associated with the digestive form, expressed as megacolon, megaesophagus, or both^8^ ^,^ ^9^. One of the most puzzling aspects of CD is determining which factors affect disease evolution, in particular, which patients will develop heart failure (HF), the severe form of Chagas heart disease with a poor prognosis^10^. More than 100 years after the discovery of CD, accurate prognosis markers are still needed. Several longitudinal studies incorporating clinical and laboratory variables have helped to define prognostic markers in cardiac syndromes^9^ ^,^ ^11^ ^-^ ^13^. In a systematic review that evaluated predictors of mortality in CD, left ventricular dysfunction, New York Heart Association (NYHA) functional classes III and IV, cardiomegaly, and nonsustained ventricular tachycardia (using a 24-hour Holter) indicate a poorer prognosis^13^. Additionally, as in other chronic cardiac diseases, molecular and genetic markers have been studied in CD based on the possibility that they may contribute to the development of Chagas heart disease^14^. However, controversial data were obtained, and so far, there is no consensus on the association of genetic biomarkers^15^ ^,^ ^16^. There were no significant results demonstrated in analyzed gene variants of cytokine-related molecules related to the development or severity of Chagas heart disease^17^.

The activation of the renin-angiotensin-aldosterone (RAAS) system is closely related to deterioration in cardiac function and HF and is among the earliest altered systems in the disease process. In this condition, the deregulation of the elements involved in the RAAS system, such as angiotensinogen, angiotensin-converting enzyme (ACE), and angiotensin-II is notable^18^. ACE polymorphisms are associated with the risk of HF^19^, mortality^20^, and unfavorable echocardiographic evolution in cardiomyopathies from diverse etiologies^19^ ^-^ ^21^. The most studied polymorphism of ACE corresponds to an insertion (I)/deletion (D) of 287 base pairs (bp) located in intron 16 and variants known as DD, ID, and II have been associated with plasma ACE activity^18^. The DD genotype, in particular, is associated with high ACE activity^22^ ^-^ ^23^, which is also related to the increased activity of angiotensin II, a key player in the pathophysiology of HF^24^ ^-^ ^25^and heart fibrosis^26^.

ACE inhibitors have demonstrated efficacy in the treatment of CD^27^. The association of the ACE variants DD, ID, and II and clinical evolution were tested in a cohort of patients with CD from Venezuela. The data supported the lack of association between ACE I/D polymorphism and the progression of CD; however, the study had limitations in sample size^28^. In the present study, an urban cohort of CD in the Northeast of Brazil was staged according to the I Latin American Guideline for CD^8^, and the prevalence of ACE I/D polymorphism in the absence of heart failure was evaluated.

## METHODS

### Study design

A total of 343 individuals were enrolled in the study for four years. All patients were born in the Northeast of Brazil. Individuals were recruited at the Chagas Disease and Heart Failure Ambulatory of the Emergency Department of Pernambuco (PROCAPE)/University of Pernambuco (UPE) after screening for positive CD serology. The confirmation of CD was a serological diagnosis based on at least two positive tests, including enzyme-linked immunosorbent assay (ELISA), Western-blotting, indirect immunofluorescence, or both, performed by the Central Reference Laboratory (LACEN) of Pernambuco, Brazil. The included patients were staged according to clinical features after anamnesis, and electrocardiogram (ECG) and echocardiogram (ECHO) results. For clinical classification, we adopted the I Latin American Guideline for diagnosis and treatment of CD cardiopathy^8^. According to the analyzed criteria, the patients were staged as follows: **stage A**, 100 patients without cardiac symptoms and normal registers on ECG and ECHO; **stage B1**, 144 patients with no clinical signs of HF, but ECG or ECHO changes such as segmental dysfunction, and normal ventricular function; and **stage C**, 99 patients with clinical signs of HF, ECG abnormalities, and structural cardiomyopathy by ECHO evaluation. Patients presenting with the digestive form of CD, co-infections, and alcohol use were excluded from the present study. The Ethics Committees of Fiocruz/RJ (License 541/09), PROCAPE/UPE (License 80210/10), and University of São Paulo (regular meeting on January 17, 2012) approved all procedures. All patients were fully informed about the study and signed informed consent forms.

### Clinical and complementary evaluation

A refereed physician evaluated all patients using their clinical history and a physical examination. HF was defined using Framingham criteria^29^ with two major or one major and two minor criteria. All patients underwent 12-lead ECG. Any changes, such as AV block, bundle-branch block, or arrhythmias, were considered to be due to CD. Left ventricular ejection fraction and echocardiography was performed by a physician blinded to the protocol using Vivid 3 (General Electric - GE, EUA), and any segmental motion impairment was considered to be due to CD.

### DNA processing and genotyping

Genomic DNA was extracted from frozen blood samples using a modified precipitation salting-out technique^30^. The DNA from each sample was quantified using NanoDrop ND-1000 Spectrophotometer (NanoDrop Technologies, USA). The I/D polymorphism (rs4646994) was evaluated using conventional PCR with the primers 5’CTGGAGACCACTCCCATCCTTTCT3’ and 5’AGACTGCTTACACTACCGGTGTAG3’ followed by 2 % agarose gel electrophoresis for direct genotyping based on the following product sizes: 490 bp fragment for genotype II; two fragments, 490 bp and 190 bp, for heterozygote genotype; and 190 bp fragment for DD genotype, as previously reported^31^.

### Statistical analysis

The dependent variable was the stage of Chagas heart disease (stages A, B1, and C), according to the I Latin American Guideline for CD (Brazilian Society of Cardiology)^8^.

 Genotypic, allelic, or minor allele carrier counts were determined using direct counting. The Hardy-Weinberg equilibrium was determined using the chi-square test. The prevalence of ACE I/D polymorphism in different stages of CD, including nongenetic covariates, such as age, sex, ethnicity, level of education, income, and previous trypanocidal treatment, were analyzed using the logistic regression model. Genetic association tests were performed by unconditional logistic regression shown as odds ratio (OR) with the respective 95 % confidence interval (CI); the adjusted analysis included age, sex, and trypanocidal treatment as covariates. Analyses were performed using Statistical Package for Social Sciences (SPSS) version 18.0 for Windows and R Environment version 3.1.1. In all statistical tests, a significance level of 0.05 was adopted.

## RESULTS

All enrolled individuals were born in the Northeast of Brazil and were admitted as patients at PROCAPE/UPE after receiving positive serology tests for CD. Table 1 presents the main characteristics of the analyzed urban cohort. For simplification, we annotated the sequential results showing the comparisons between the case-control groups as B1 (case) *vs* A (control), C (case) *vs* B1 (control), and C (case) *vs* A (control). The average ages (years) among the groups are as follows: A (51 ± 12), B1 (61 ± 13), and C (59 ± 12). The comparisons for this variable between the case-control groups supported significant differences (*p* < 0.001, *p* = 0.331, and *p* < 0.001, respectively). Distribution of patients according to sex (*p* = 0.075, *p* = 0.008, and *p* = 0.430, respectively), ethnicity (*p* < 0.001, *p* < 0.001, and *p* = 0.879, respectively), education levels (*p* < 0.001, *p* < 0.001, and *p* = 0.866, respectively), and minimum wage income (*p* < 0.001, *p* < 0.001, and *p* = 0.279, respectively) also revealed differences among the three study groups.

**TABLE 1:** General and demographic characteristics of the individuals included in the study.

Variable^a^	A Group	B1 Group	C Group
	N = 100	N = 144	N = 99
**Sex**			
Female	66 (66.0)	110 (76.4)	60 (60.6)
Male	34 (34.0)	34 (23.6)	39 (39.4)
Age (years)^b^	51 ± 12	61 ± 13	59 ± 12
**Ethnicity (n = 280)**	**75**	**123**	**82**
White	20 (26.7)	21 (17.1)	19 (23.2)
Black	8 (10.7)	11 (8.9)	9 (11.0)
Mestizo	47 (62.7)	91 (74.0)	54 (65.9)
**Monthly income (n = 287)**	**94**	**107**	**86**
Up to 1 MW	62 (66.0)	88 (82.2)	66 (76.7)
2 -4	14 (14.9)	19 (17.8)	9 (10.5)
More than 5	18 (19.1)	0 (0.0)	11 (12.8)
**Education (n = 321)**	**93**	**137**	**91**
Up to 4 years	52 (55.9)	108 (78.8)	52 (57.1)
More than 4 years	41 (44.1)	29 (21.2)	39 (42.9)
**Region (n = 337)**	00	**138**	**99**
PE, Countryside	18 (18.0)	13 (9.4)	13 (13.1)
PE, Woods	16 (16.0)	23 (16.7)	25 (25.3)
PE, Metropolitan	40 (40.0)	75 (54.3)	50 (50.5)
PE, Backwoods	22 (22.0)	26 (18.8)	8 (8.1)
Other States	4 (4.0)	1 (0.7)	3 (3.0)

a Results are presented as number (%); ^b^Mean ± standard deviation; **n:** number of patients with the referred variable; **PE:** State of Pernambuco; **MW:** minimum wage.

Intrinsic to their clinical stages the left ventricular ejection fraction (LVEF) was 38.1 % ± 8.6 % in stage C patients, 65.7 % ± 6.6 % in stage B1 patients, and 66.7 % ± 4.9 % in stage A patients (*p* = 0.173, *p* < 0.001, and *p* < 0.001, respectively). Inherent to cardiac status (stage C showing HF), drugs such as ACE inhibitors or angiotensin receptor blockers, were prescribed for 85.9 % of patients in group C, 22.9 % in group B1 and 20 % in group A (*p* = 0.963, *p* < 0.001, and *p* < 0.001, respectively). Further, spironolactone was prescribed for 39 % of patients in group C, but not for patients in group B1 and group A. Importantly, use of the trypanocidal drug benznidazole was reported in 12.1 % of stage C patients, 9 % of stage B1, and 48 % of stage A (*p* < 0.001, *p* = 0.435, and *p* < 0.001, respectively).

Regarding the genetic analyses, we observed that genotypic frequencies were distributed per the Hardy-Weinberg equilibrium (*p* > 0.05) for all tested groups. Unconditional logistic regression results comparing patients included in stage B1 (case) compared with patients in stage A (control) are detailed in Table 2. No significant differences were found for either genotype, allelic, or carrier comparisons. We then compared the stage C (case) patients with stage A (control) patients, as shown in Table 3. In stage C patients compared with those in stage A, genotypes DI or DD of the *ACE* I/D polymorphism was more prevalent in patients with heart failure; *p* = 0.02, DI genotype: OR = 2.52 [CI = 1.13 - 5.59] and DD genotype: OR = 2.59 [CI = 1.12 - 5.95]. The association was consistent with the increased number of individuals in the C population exhibition who exhibited DI (51 % in C *vs.* 43 % in A) and DD (37 % in C *vs.* 31 % in A) genotypes. However, after statistical adjustments for sex, age, and use of the trypanocidal drug benznidazole, the association was found to be borderline for DD genotype (*p* = 0.06) and D carriers (*p* = 0.06).

**TABLE 2:** Genotypic, allelic, and carrier frequencies for ACE I/D (rs4646994) polymorphism. Logistic regression results comparing Chagas disease patients of Group B1 with Group A.

	Group B1	Group A	Odds Ratio	95% CI	P value	Odds Ratio^a^	95% CI^a^	P value^a^
	N = 144	N = 100						
	(control)	(control)						
II	30 (0.21)	26 (0.26)			- Reference -			
DI	68 (0.47)	43 (0.43)	1.37	0.72 - 2.62	0.34	1.06	0.51 - 2.42	0.80
DD	46 (0.32)	31 (0.31)	1.29	0.64 - 2.58	0.48	1.11	0.51 - 2.20	0.87
I Allele	128 (0.44)	95 (0.48)			- Reference -			
D Allele	160 (0.56)	105 (0.52)	1.13	0.68 - 1.89	0.64	1.06	0.60 - 1.86	0.85
D Carriers	114 (0.79)	74 (0.74)	1.34	0.73 - 2.44	0.35	1.08	0.55 - 2.12	0.82

Results are presented as N (frequency). Overall P value for genotype ANOVA= 0.63. ^a^OR values are corrected for the nongenetic variables sex, age, and the use of trypanocidal treatment. **n:** number of patients; **CI:** confidence interval.

**TABLE 3:** Genotypic, allelic, and carrier frequencies for *ACE* I/D (rs4646994) polymorphism. Logistic regression results comparing Chagas disease patients in Group C with those in Group A.

	Group C	Group A	Odds Ratio	95% CI	P value	Odds Ratio^a^	95% CI^a^	P value^a^
	N = 99	N = 100						
	(case)	(control)						
II	12 (0.12)	26 (0.26)			- Reference -			
DI	50 (0.51)	43 (0.43)	2.52	1.13 - 5.59	0.02	2.02	0.88 - 4.70	0.10
DD	37 (0.37)	31 (0.31)	2.59	1.12 - 5.95	0.02	2.32	0.96 - 5.60	0.06
I Allele	74 (0.37)	95 (0.48)			- Reference -			
D Allele	124 (0.63)	105 (0.52)	1.52	0.86 - 2.67	0.15	1.06	0.60 - 1.86	0.85
D Carriers	87 (0.88)	74 (0.74)	2.55	1.20 - 5.40	0.02	2.15	0.97 - 4.73	0.06

Results are presented as N (frequency). Overall P value for genotype ANOVA = 0.04. ^a^OR values are corrected for the nongenetic variables sex, age, and the use of trypanocidal treatment. **CI:** confidence interval.

The comparison of stage C (case) patients with stage B1 (control) patients also indicates that the frequencies of DD and DI genotypes are slightly higher in the more severe clinical form. However, *p*-values were not significant (Table 4). Lastly, to challenge the association of *ACE* I/D polymorphism with the progression of heart failure in CD, patients with positive CD serology in stage C (case) HF were compared with patients with positive CD serology in stages A and B1 without HF combined into one group (control). As shown in Table 5, our results indicate that after corrections for sex, age, and use of the trypanocidal drug benznidazole, DD genotype (OR = 2.09; CI = 0.99-4.40; *p* = 0.05) and D carriers (OR = 2; CI = 1.01-3.96; *p* = 0.04) were more prevalent in patients with HF due to CD.

**TABLE 4:** Genotypic, allelic and carrier frequencies for *ACE* I/D (rs4646994) polymorphism. Logistic regression results, comparing Chagas disease patients in Group C with those in Group B1.

	Group C	Group B1	Odds Ratio	95% CI	P value	Odds Ratio^a^	95% CI^a^	P value^a^
	N = 99	N = 144						
	(case)	(control)						
II	12 (0.12)	30 (0.21)			- Reference -			
DI	50 (0.51)	68 (0.47)	1.84	0.86 - 3.94	0.12	1.78	0.82 - 3.87	0.14
DD	37 (0.37)	46 (0.32)	2.01	0.91 - 4.46	0.09	1.95	0.87 - 4.37	0.11
I Allele	74 (0.37)	128 (0.44)			- Reference -			
D Allele	124 (0.63)	160 (0.56)	1.34	0.79 - 2.26	0.27	1.32	0.78 - 2.24	0.30
D Carriers	87 (0.88)	114 (0.79)	1.90	0.92 - 3.94	0.08	1.85	0.89 - 3.86	0.10

Results are presented as N (frequency). Overall P-value for genotype ANOVA= 0.19. ^a^OR values are corrected for the nongenetic variables, sex, age, and the use of trypanocidal treatment. **CI:** confidence interval.

**TABLE 5:** Genotypic, allelic, and carrier frequencies for *ACE* I/D (rs4646994) polymorphism. Logistic regression results comparing Chagas disease patients of Group C with the combined groups; A and B1.

	Group C	Group A + B1	Odds Ratio	95% CI	P value	Odds Ratio^a^	95% CI^a^	P value^a^
	N = 99	N = 244						
	(case)	(control)						
II	12 (0.12)	56 (0.23)			- Reference -			
DI	50 (0.51)	111 (0.45)	2.10	1.03 - 4.26	0.04	1.94	0.95 - 3.98	0.07
DD	37 (0.37)	77 (0.32)	2.24	1.07 - 4.68	0.03	2.09	0.99 - 4.40	0.05
I Allele	74 (0.37)	223 (0.46)			- Reference -			
D Allele	124 (0.63)	265 (0.54)	1.41	0.87 - 2.28	0.16	1.36	0.84 - 2.21	0.21
D Carriers	87 (0.88)	188 (0.77)	2.16	1.10 - 4.23	0.02	2.00	1.01 - 3.96	0.04

Results are presented as N (frequency). Overall P-value for genotype ANOVA = 0.06. ^a^OR values are corrected for the nongenetic variables, sex, age, and the use of trypanocidal treatment. **CI**: confidence interval.

## DISCUSSION

Herein, we provide evidence supporting the higher prevalence of DD genotype/D carriers of *ACE* I/D polymorphism in HF due to Chagas disease in a case-control study of a population from Northeast Brazil. It has been suggested that the DD genotype is associated with higher serum angiotensin (s-ACE) activity and, consequently, the conversion of angiotensin I to angiotensin II in different populations^22^ ^,^ ^23^. The genotype/phenotype correlation has been consistently associated with inflammation and tissue injury that, in turn, regulates cardiovascular risks, such as stroke or inflammatory diseases^19^ ^-^ ^23^. Although our cohort comprises a small number of patients in each stage and our study lacks a confirmatory functional analysis, the results are consistent and report an increased frequency of DD genotype among stage C patients compared with stages A or B1 patients. Intronic D/I *ACE* polymorphism has been an important tag to the region, and several other single nucleotide polymorphisms in coding and promoter regions are in linkage disequilibrium, which might explain the functional association of this polymorphism with s-ACE activity levels^22^ ^,^ ^23^ ^,^ ^32^.

The population analyzed in our study has social and economic characteristics common to most of the populations afflicted with CD, a neglected tropical disease associated with poverty^2^. Independent of the group categorized by clinical features (A, B1, and C), most of the patients (66-82.2 %) have a low income (including minimum wage; US$250-300/month). Furthermore, the majority of patients (55.9-78.8 %) had a low level of education (up to 4 years). The clinical characteristics of our study group and intervening variables were typical of most CD cohorts, including sex, age, previous use of a trypanocidal drug, and use of a cardioprotective medication^33^. This indicates that the suitability of this recruitment to test the hypothesis of the *ACE* I/D polymorphism was higher in patients with CD and severe forms of cardiac disease with HF. However, a limitation was the number of study patients and the length of follow up. 

Since the 1990s, several RAA system polymorphisms have been identified, including the I/D polymorphism of the ACE gene^22^. The DD genotype is associated with increased activity of the ACE enzyme in the myocardium^34^. Therefore, this finding helped to predict that the D allele is associated with diseases involving increased activity of the RAA system. However, the association of *ACE* I/D polymorphism with cardiovascular diseases is rather controversial. A study found no association between the I/D polymorphism and HF^35^. Conversely, cardiac hypertrophy^36^ and higher heart weight^37^ were associated with the DD genotype. A meta-analysis suggested that there was an association between stroke and the *ACE* genotype^38^, indicating that DD carriers have a higher risk of stroke. Additionally, a more recent meta-analysis supports that the D allele is associated with an increased risk of ischemic stroke in Asians, but not in Caucasians^39^.

In the natural history of CD, ~30 % of the chronically *T. cruzi*-infected patients evolve to the cardiac form of the disease, which shows a wide-ranging presentation, from mild to severe with HF, and a poor prognosis^13^. In CD, therapeutic response to ACE inhibitors has beneficial effects^27^. Therefore, it was logical to explore a putative association of the *ACE* I/D polymorphism with the progression of the cardiac form of CD. Regarding CD, the only published study to evaluate the association of the ACE gene with the development of heart damage was conducted in a Venezuelan cohort. Although the sample size was limited, and the analysis was not statistically significant, the results suggest that the progression of chagasic cardiomyopathy was unrelated to the I/D polymorphism^28^, which is difficult to conclude. Although the sample size is our study limitation, it was conducted in a larger population than the Venezuelan study. It was stratified by disease severity, demonstrating a higher prevalence of ACE I/D polymorphism in the severe form of heart disease with HF.

The D allele of the ACE gene occurs in approximately 55 % of the healthy population^34^. In our study, the frequencies of the D allele of the ACE gene were 52 % in stage A patients, 56 % in stage B1 patients, and 63 % in stage C patients. A possible association of the *ACE* D allele with advanced stages of other cardiomyopathies from diverse etiologies has also been reported^19^ ^,^ ^21^ ^,^ ^40^. Furthermore, various studies have reported a relationship between the plasma activity of ACE and the individual genotype. Thus carriers of the DD genotype are said to have the highest ACE concentrations, while individuals with homozygote genotype II are suggested to have the lowest concentrations^23^ ^,^ ^41^ ^,^ ^42^. It is estimated that the D allele contributes approximately half of the variation in plasma levels of ACE^43^. Despite some disagreement, the DD genotype has been associated with an increased risk of HF and mortality^21^. In a cohort study, an evaluation of idiopathic HF patients demonstrated that the DD genotype remained as a predictor of death in multivariate analysis, which suggests the importance of this genotype profile as an influential factor in reducing survival among HF patients^20^. Our results are consistent with the higher prevalence of DD genotype/D carriers in patients with a more severe cardiac form of CD. Despite the study limitations regarding sample size (convenience) and follow up, perhaps prophylactic treatment in this group of patients should be considered. Indeed, a previous study showed that pharmacotherapy might mitigate the deleterious effects of the DD genotype, which may be expressed in the absence of medications^44^.

Possible pharmacotherapy interaction with the *ACE* I/D polymorphism has also been discussed. In a cohort study of patients with systolic HF, the D allele was associated with an unfavorable evolution; the impact was solely observed in the group not treated with ACE inhibitors and beta-blockers^45^, suggestive of potential interaction between the I/D polymorphism and the therapy. In our study, 88 % of patients in stage C were prescribed the neurohormone blockers: beta-blockers, angiotensin-converting enzyme inhibitors or angiotensin II receptor blockers, and mineralocorticoid receptor antagonists at the maximum dose tolerated. However, a few patients in this stage did not use any of these medications. Furthermore, in our study, the influence of I/D polymorphism in Chagas heart disease progression may be masked by the use of the cardioprotective medications ACE/ARB inhibitors, also prescribed for 20 % of the patients in stage A and 22.9 % of the patients of stage B1. Silva et al^46^ recently published a study developed in Goias - Brazil, with no differences in the distribution of (Insertion/Deletion) genotype frequencies of ACE polymorphism regarding the severity of Chagas heart disease. There were, however, several differences between the evaluation methods, including the inclusion criteria such as ventricular dysfunction, geographic differences, and the sample size. Moreover, our study had more strict clinical and laboratory criteria. Despite no strong evidence of the benefit regarding trypanocidal treatment, it may have affected our results as 48 % of stage A patients were given this treatment compared to 12.1 % of stage C. We minimized this confounder by adjusting the results for the trypanocidal treatment. In summary, our results suggest that the *ACE* I/D polymorphism is more prevalent in patients with the cardiac form of Chagas disease and HF in this case-control study comprising a population from the Northeast region of Brazil.
